# Hong Kong Chinese Medicine Clinical Practice Guideline for Cancer Palliative Care: Pain, Constipation, and Insomnia

**DOI:** 10.1155/2019/1038206

**Published:** 2019-01-22

**Authors:** Wai Ching Lam, Linda Zhong, Yuqi Liu, Nannan Shi, Bacon Ng, Eric Ziea, Zhaoxiang Bian, Aiping Lu

**Affiliations:** ^1^Hong Kong Chinese Medicine Clinical Study Centre, School of Chinese Medicine, Hong Kong Baptist University, Kowloon 999077, Hong Kong; ^2^China Academy of Chinese Medical Science, Beijing 100700, China; ^3^Department of Chinese Medicine, Hong Kong Hospital Authority, Kowloon 999077, Hong Kong

## Abstract

It is common for patients with cancers in Hong Kong seeking Chinese Medicine (CM) therapies as supportive care during cancer treatment and to manage treatment-related side effects. This article provides clinical practice guideline (CPG) on the use of CM for specific clinical indications caused by cancer and during cancer treatment, including pain, constipation, and insomnia, and aims to guide local licensed CM practitioners and provide beneficial reference for social medical decision makers and patients. In this manuscript, we summarize the clinical manifestation, CM pattern classification, and CM intervention including herbal treatment, acupuncture treatment, regulating, and nursing based on pattern differentiation.

## 1. Introduction

According to the most update statistics from the Hong Kong Government, in 2015, number of new cases of cancer in Hong Kong hit a historical high of 30,318 and 14,316 deaths which were attributable to cancers [1]. Multiple studies have pointed out that a significant proportion of cancer patients with moderate to severe pain, nausea, constipation, insomnia, anxiety, and other symptoms can benefit from palliative care [2–4]. Suffering from the symptoms, patients in Hong Kong commonly seek help from complementary and integrative therapies, especially CM, as supportive care during cancer treatment and manage treatment-related side effects [2].

Since CM is well known for its clinical effect in improving the quality of life of cancer patients, in 2014, the Hospital Authority (HA) has been commissioned by the Hong Kong Government to launch a project named “Integrated Chinese-Western Medicine Pilot Programme” [5]. Eligible in-patients at the designated hospitals started receiving CM treatment based on clinical protocol developed by Chinese and Western Medicine experts. The pilot programme aims to explore feasibility and gain experience via small-scale pilots in order to explore the development of an Integrated Chinese-Western Medicine (ICWM) treatment model. Therefore, in order to standardize the diagnosis and treatment with CM and provide evidence-based clinical references for both Chinese and Western physicians, the Hong Kong Baptist University is entrusted by the HA to complete the CM CPG development process. The guideline development team, based on thorough analysis of the CM diagnosis and treatment environment in Hong Kong, adopted evidence-based medicine, qualitative research, and text mining techniques to provide the best evidence for the guidelines and completed the development of the guidelines. The guideline development team is based on the principle of multidisciplinary integration. Its members include health care policy makers, medical experts, methodologists, literature experts, and patient representatives.

Unlike other clinical guideline recommendations suggested to be used as the standard of therapy, we use the term “recommendation” to conclude CM treatments should be considered as a viable but not singular option for the management of a specific CM pattern. As CM contributes tailoring treatment based on symptom differentiation, combination-based approaches and the interactions of the numerous permutations are possible and still under investigating, such that recommendations must account for this limitation of our knowledge. Despite these limitations to evaluating the use of CM therapies in the oncology setting, the models provide sufficient evidence to warrant recommendations on the therapies as viable options for treating specific conditions.

In this article, we introduce the formation, promotion, execution, and update of the CPG in brief and then provide background for each CM therapy that has a sufficiently large body of evidence and historical support to formulate the recommendations. Information is also provided for CM patterns diagnosis criteria and intervention details and to implement the recommendations into the different CM patterns, respectively, for cancer-related pain, constipation, and insomnia.

The goal of this study is to summarize the methods and measures for the treatment of CM on pain, constipation and insomnia in cancer palliative care and to make a reasonable evaluation by careful review of existing practice and available published evidence on topic, in order to promote it and provide benefits for social medical decision makers and patients.

Data sources are labeled following by major content items. Sources from text mining are indicated by “*※*” and sources from Hong Kong experts' consensus are labeled by “§”. Levels of evidence and classes of recommendations are directly labeled after literature evidence.

## 2. Methods

### 2.1. Data Query and Download

This study was conducted according to our previous work [29]. To avoid repetition of clinical study and ensure efficiency, we chose SinoMed database as data source and identified clinical researches with CM treatment for cancer palliative care in SinoMed, which is the most comprehensive electronic medicine database in China. The time range is from 1979 to 1 January 2015. The following search terms were used: “cancer AND pain” OR “cancer AND insomnia” OR “cancer AND constipation” OR “cancer AND lymphedema” OR “cancer AND anorexia” OR “cancer AND lethargy” (in Chinese). As a result, 197213 records for cancer palliative care were retrieved.

### 2.2. Data Mining Process

After retrieving data from SinoMed, we listed the data order by download order. In order to transfer the interesting data into the framework of structured file system, a tool has been developed (software copyright with submitted ID 0261882 and registered ID 2010SR073409) to transfer its plain TXT data into Microsoft® SQL Server® 2008 R2 Enterprise Edition. Then we use a data slicing algorithm “discrete derivatives” based on the calculation of frequency with detailed algorithm described in our previous research [30], to filter high frequencies of (1) CM pattern classification and symptom differentiation; (2) herbal decoction; (3) acupuncture treatment. The data mining process was deployed on source data collected above with cancer and specified symptoms.

### 2.3. Existing Clinical Evidence Searching

With searching strategy, a combination of electronic and manual screening was adopted for systematic reviews of CM treatment on relevant symptoms. Cochrane guidelines were applied for methodological evaluation of literature quality. And we used the grades of recommendations and the levels of evidence proposed by Professor Jianping Liu of China designed for traditional medicines [31].

### 2.4. Hong Kong Local Expert Consensus

On the key issues in the CPG, Delphi survey was applied to collect professional advice from licensed CM practitioners in Hong Kong based on the literature searching and documented clinical evidences. After 2 rounds of surveys, Hong Kong local experts obtained consensus opinions on the contents of the items which will serve as one of the main sources for the formation of CPG.

### 2.5. Data Synthesis

Information from three aspects, (1) literature hotspots; (2) existing clinical evidence; (3) local expert consensus, is summarized and listed for optional items in CPG. Items with insufficient data support were excluded.

### 2.6. Review and Consultation

A comprehensive review of the CPG was conducted through an expert consensus meeting and finalized the items for inclusion in the CPG.

### 2.7. Drafting CPG

The guideline development group developed a framework and drafted the CPG accordingly.

### 2.8. Promotion

The electronic version of this study will be published on the official websites of the HA and the Hong Kong Registered Chinese Medicine Practitioners Association. Also, publicity and promotion efforts will be conducted to Hong Kong CM practitioners.

### 2.9. Execution

It is the first time guidelines for CM clinical practice on cancer palliative care are formulated in Hong Kong. The production of this guide is just a beginning and an attempt. More experience and feedback are needed for further summarization.

### 2.10. Update

CPG development panel will regularly entrust relevant professional personnel to have review through collecting, collating, and analyzing newly emerged evidence. CPG development panel has the responsibility and rights for decisions on the revision. In general, the CPGs need to be revised or updated in the following cases: (1) to include new intervention methods; (2) to provide evidences prove that the existing intervention methods are the best, beneficial, or detrimental; (3) in adding new important or meaningful conclusions; (4) in generating new medical resources.

## 3. Main Results

### 3.1. Definition

#### 3.1.1. CM for Cancer Pain

Cancer pain is a subjective, multidimensional symptom that may affect patients throughout and after the course of the disease and its treatment [32]. It is estimated that one-quarter of cancer patients experience cancer pain at diagnosis, one-third during treatment, and three-quarters during advanced stages [33, 34]. If the cancer pain is not relieved, the patient will feel extremely uncomfortable and may cause fatigue, anxiety, depression, loss of appetite, and other symptoms, and seriously affect the patient's daily activities, self-care ability, and overall quality of life [35]. Cancer pain has long been recorded for in ancient CM literatures since Han dynasty with various descriptions and naming on the symptoms. Moreover, many different evidences from reviews and clinical trials have showed that CM treatments were associated with cancer pain relief as well as improving quality of life [36–42].

#### 3.1.2. CM for Cancer Constipation

Constipation is the third common symptom in palliative care cancer patients, only superseded by pain and anorexia [43–45]. It is important to be mentioned that constipation patient groups can be divided into defecation disorders which do not respond to laxatives but cognitive behavioral therapy and opioid induced constipation response to peripherally acting mu-opioid receptor antagonists (PAMORAs) and linaclotide and lubiprostone [45–47]. According to the clinical practice guidelines, a combination of a stimulant (e.g., senna or sodium picosulphate) and an osmotic laxative (e.g., polyethylene glycol or lactulose) is generally recommended [47]. However, over 60% of patients prescribed laxatives reported certain degrees of constipation (e.g., inadequate pushing force, sense of incomplete defecation, or difficult defecation) [48]. Therefore, CM become an alternative choice for researchers and cancer patients. According to the CM theory, constipation can be broadly divided into different patterns based on the underlying aetiology and severity [49]. Similar to cancer pain, evidences present in CM herbal, acupuncture, regulating, and nursing treatments in form of reviews and clinical trials [50–55].

#### 3.1.3. CM for Cancer Insomnia

Insomnia is one of the most common symptoms experienced by cancer patients before, during and after cancer-related treatment with prevalence rates of 30%-70% [56–63]. And it has significant correlation to psychological distress, reduces physical functioning, and impairs quality of life in cancer patients [64–66]. Although insomnia has high prevalence and negative consequences, it is often neglected and undertreated [67]. In CM theory, treatments can be designed to treat the full range of physical and emotional disorders because they are interlocking each other. Currently, evidence supports the use of CM herbal treatment in cancer therapy related insomnia in both cancer patients and survivors [68]. In addition, emerging researches suggest that, not limited to herbal medicine, acupuncture and nursing are also useful for treatment of commonly occurring cancer-related psychological symptoms [54, 69, 70]. As a result, patients and providers alike are interested in the evidence-based nonpharmacologic alternatives such as CM therapy for these symptoms [71].

### 3.2. Clinical Manifestation

CPG development panel consulted the patterns classification based on (1) literature hotspots; (2) existing clinical evidence; (3) local expert consensus and then determined 6 common CM patterns of cancer pain, 4 common CM patterns of cancer constipation, and 5 common CM patterns of cancer insomnia (Tables 1–4).

With the classification of patterns above, clinical CM practitioners can refer to the recommended pattern-related treatment methods. Based on further symptom differentiation and combine diagnosis, CM practitioners can tailor best-fit therapy for every patient.

## 4. CM Intervention

### 4.1. Herbal Treatment (Tables 5–7)

CM herbal medicine is applied commonly in intervention of cancer pain, constipation, and insomnia which can also cooperate with acupuncture or other comprehensive treatment options such as regulation and nursing. For patients with concurrent patterns and presented as complicated patterns, CM contributes tailoring treatment based on symptom differentiation; therefore, combination-based approaches can be considered.

In daily practice, patterns of pain, constipation, and insomnia are not completely consistent to the descriptions in defined CM patterns. Therefore, cross reference should be consulted. It is recommended that prescription should be on the base of comprehensive consideration of type of cancer, pattern, and symptom. For instance, pattern of qi movement stagnation is one of the most common cancer-related pain patterns in clinical. If patient complicates with symptoms of phlegm and blood stasis on this basis, the pattern could be treated as qi movement stagnation in coordination with combination of phlegm and blood stasis pattern; moreover, modified diagnosis is recommended based on the cancer type, characteristics of qi, phlegm, blood, and pain symptom. When clinical effects are not optimal, the relationship among various points of pattern differentiation such as deficiency and excess, cold and heat, qi and blood, dredging and descending, and pathological products should be comprehensively taken into account. Besides, other patterns differentiation methods, for example, microcosmic pattern differentiation, could be utilized to search possible reasons.

According to the yin and yang sleep theory of CM, the yang qi rises during the daytime and reaches apex at noon. Combined with the understanding of modern time biology and clinical experience, it is recommended to follow a medicine taking approach stated by an ancient CM practitioner in Ming dynasty that insomnia patients are required to take the medicine one hour after lunch and one hour after dinner (grading of recommendation: C^§^; level of evidence: IV).

### 4.2. Acupuncture Treatment (Tables 8–10)

Acupuncture is another treatment option to treat a variety of symptoms and conditions associated with cancer and the side effects of cancer treatments. It is a family of procedures involving stimulation of anatomical locations on the skin by a variety of techniques. The most studied mechanism of stimulation of acupuncture points uses penetration of the skin by thin, solid, metallic needles, which are manipulated manually or by electrical stimulation [72].

Acupoints recommended for treatment of lung cancer pain are He Gu (LI4), Nei Guan (PC6), and Kong Zui (LU6), for treatment of liver cancer pain are He Gu (LI4), Nei Guan (PC6), Yang Ling Quan (GB34), and Zhong Du (LR6), and for treatment of intestinal cancer are He Gu (LI4), Nei Guan (PC6), Zhong Wan (RN12), Zu San Li (ST36), and Zhi Gou (SJ6) (grading of recommendation: B^*※*§^; level of evidence: IIa).

### 4.3. Regulating and Nursing

#### 4.3.1. Regulating on Cancer Pain

Herbal dishes, static qigong, qigong exercise, ointment or medicinal oil for external use, bloodletting, auricular point (auricular beads), cold compress or hot compress, moxibustion, emotional counseling, sufficient sleep, etc. can be used to relieve cancer pain (grading of recommendation: C^§^; level of evidence: IV).

#### 4.3.2. Regulating on Cancer Constipation

Develop healthy eating habits, to eat mostly foods derived from vegetables, fruits, and whole grains, often to hydrate and limit spicy, highly fatty, or processed foods. Avoid excessive drinking or eating too much cold food. Strengthen regular physical exercise and avoid sedentary activities, develop regular bowel habits, avoid excessive mental stimulation, and maintain a pleasant mood. For elderly and chronic constipation patients, treating with enema or other external treatments can prevent spending a lot of time on the toilet straining which might induce acne, hematochezia, and even angina pectoris, myocardial infarction, or other symptoms (grading of recommendation: C^§^; level of evidence: IV).

#### 4.3.3. Prevention and Nursing on Cancer Insomnia

In aspect of prevention, CM theory stated that insomnia is due to disorder of brain function. Therefore, it is important to promote a healthy brain and mind by maintaining pleasant mood and healthy work-life balance (grading of recommendation: B^*※*§^; level of evidence: IIa/IIIb) [73].

In aspect of nursing, it is to cooperate with CM practitioners in the treatment and care of elderly and disabled patients. Nursing includes basic care and specialist care. Basic nursing work includes daily life care, primary diagnosis and treatment techniques, daily observation, nutrition, disinfection and isolation, and hygiene, in order to establish proper environment to relief insomnia. There should be time for mind relaxing before going to sleep, avoiding spending time on worrying about falling asleep, and avoiding alcohol, caffeine, and nicotine. Appropriate relief exercises should be added after dinner. There is no tea or coffee before going to bed. Turn off the sound while sleeping, pull the curtains down, turn off the lights, and develop good sleep habits. Take Chinese Medicine on time to promptly treat related diseases. On sleep posture, generally, right lateral recumbent position is adopted while supine and prone positions should be avoided. In diet, take diet and health foods that promote sleep. Finally, methods such as medicine pillow, massage, and herbal dishes are also recommended for insomnia patients (grading of recommendation: B^*※*§^; level of evidence: IIIb) [73].

## Figures and Tables

**Table 1:** Common CM patterns of cancer pain, constipation, and insomnia.

**Cancer associated symptom**	**Common CM pattern classification**
Pain	(1) Qi movement stagnation
	(2) Phlegm-dampness congealing
	(3) Static blood obstructing
	(4) Qi stagnation and blood stasis
	(5) Combination of phlegm and blood stasis
	(6) Qi and blood deficiency

Constipation	(1) Qi and yin deficiency
	(2) Qi movement stagnation
	(3) Blood deficiency
	(4) Yang deficiency

Insomnia	(1) Liver-qi stagnation
	(2) Liver depression and spleen deficiency
	(3) Qi and blood deficiency
	(4) Qi and yin deficiency
	(5) Heart and spleen deficiency

**Table 2:** Diagnostic criteria of CM patterns of cancer pain.

**Pattern**	**Diagnosis**
Qi movement stagnation^*※*§^	Abdominal/chest pain, migratory pain, often aggravated by bad mood, pale tongue with white fur, stringy pulse

Phlegm-dampness congealing^*※*§^	Cough with shortness of breath, chest/hypochondriac pain, epigastric fullness sensation, cough and spitting inducing pain, unable to supine due to cough and shortness of breath, abdominal distension as drums, feeling heavy dampness like wrap, unbearable abdominal pain, pale or pink tongue with white and greasy fur, slippery pulse

Static blood obstructing^*※*§^	Stabbing pain, fixed pain, more severe at night, dark purple tongue with static blood spots and white fur, astringent pulse

Qi stagnation and blood stasis^*※*§^	Pain, or severe pain, breast tenderness in women, sunken or stringy pulse

Combination of phlegm and blood stasis^*※*§^	Pain, difficulty swallowing, dizziness, greasy fur, stringy pulse.

Qi and blood deficiency^*※*§^	Pain, dizziness, fatigue, white fur, sunken pulse

**Table 3:** Diagnostic criteria of CM patterns on cancer constipation.

**Pattern**	**Diagnosis**
Qi and yin deficiency^*※*§^	Without dry stool, or with dry stool like nuts, spend a lot of time on the toilet straining, shortness of breath, fatigue, flushed cheeks, dizziness with tinnitus, emaciation, relative weakness in the loins and knees

Qi movement stagnation^*※*§^	Difficult defecation, with or without dry stool, frequent eructation, fullness and pain in abdomen/hypochondrium, thin and greasy fur, stringy pulse.

Blood deficiency^*※*§^	Dry stool, pale white complexion, palpitation and forgetfulness, dizziness

Yang deficiency^*※*§^	With or without dry stool, difficult defecation, clear abundant urine, pale white and greenish complexion, cold limb, with preference for warmth and sensitive to cold, cold and pain in the abdomen, feeling of cold and heaviness in the loins and along spinal

**Table 4:** Diagnostic criteria of CM patterns on cancer insomnia.

**Pattern**	**Diagnosis**
Liver-qi stagnation^*※*§^	Insomnia, constipation, pain, white fur, stringy pulse, or fullness and discomfort in chest/hypochondrium, susceptible sigh

Liver depression and spleen deficiency^*※*§^	Insomnia, fatigue, pain, white fur, stringy pulse, or fullness and discomfort in chest/hypochondrium, anorexia, sallow complexion

Qi and blood deficiency^*※*§^	Insomnia, fatigue, hair loss, dizziness, white fur or red tongue with thin fur, thready or weak pulse, feverish palms and soles, night sweats, thirst, shortness of breath with lassitude

Qi and yin deficiency^*※*§^	Insomnia, fatigue, pain, dizziness, constipation, sunken or thready pulse

Heart and spleen deficiency^*※*§^	Sleepy, unable to have deep sleep, unsound slumber, difficult to fall back asleep after waking up, palpitation, forgetfulness, general lassitude, anorexia, sallow complexion, flat feeling in mouth with tasteless, abdominal distension after eating, pink tongue with white fur, thready or weak pulse

**Table 5:** Herbal treatment on cancer pain.

**Pattern**	**Pathogenesis and treatment principle**	**Recipe and ingredient herbs**	**Grading and level**
Qi movement stagnation	Pathogenesis: Liver qi depression, qi depression and blood stagnation Principle: Soothe the liver and regulate qi, activate blood to relieve pain	Modified Chai Hu Shu Gan San: Chai Hu (Bupleuri Radix), Qing Pi (Citri Reticulatae Pericarpium Viride), Chen Pi (Citri Reticulatae Pericarpium Viride), Ba Yue Zha (Fruit of Fiverleaf Akebia), Wu Yao (Linderae Radix), Xiang Fu (Cyperi Rhizoma), Chuan Lian Zi (Toosendan Fructus), Hou Pu (Magnoliae Officinalis Cortex), Yan Hu Suo (Corydalis Rhizoma), Zhi Shi (Aurantii Fructus Immaturus), Bai Shao (Paeoniae Radix Alba), Fo Shou (Citri Sarcodactylis Fructus) Modified Si Ni San: Chai Hu (Bupleuri Radix), Bai Shao (Paeoniae Radix Alba), Zhi Shi (Aurantii Fructus Immaturus), Gan Cao (Glycyrrhizae Radix Et Rhizoma)	Grading of recommendation: A^*※*§^ Level of evidence: Ib [6]

Phlegm-dampness congealing	Pathogenesis: Water-fluid retention, qi movement stagnation Principle: Purge the lung to remove water, direct qi downward to relieve pain	Modified Ting Li Da Zao Xie Fei Tang: Ting Li Zi (Descurainiae Semen Lepidii Semen), Bai Jie Zi (White Mustard Seed), Ban Xia (Pinelliae Rhizoma), Zhe Bei Mu (Fritillariae Thunbergii Bulbus), Dan Nan Xing (Arisaema Cum Bile), Kun Bu (Laminariae Thallus Eckloniae Thallus), Gua Lou (Trichosanthis Fructus), Huang Yao Zi (Dioscorea Bulbifera), Da Zao (Jujubae Fructus), Chen Pi (Citri Reticulatae Pericarpium Viride)	Grading of recommendation: C^§^ Level of evidence: IV

Static blood obstructing	Pathogenesis: Static blood retention, meridians and collaterals obstruction Principle: Activate blood and resolve stasis, dispel stasis to relieve pain	Modified Tao Hong Si Wu Tang: Dang Gui (Angelicae Sinensis Radix), Chi Shao (Paeoniaeradix Rubra), Chuan Xiong (Chuanxiong Rhizoma), Dan Shen (Salviae Miltiorrhizae Radix Et Rhizoma), Yan Hu Suo (Corydalis Rhizoma), San Qi (Notoginseng Radix Et Rhizoma), Ru Xiang (Olibanum), Mo Yao (Myrrha) Modified Fu Yuan Huo Xue Tang: Chai Hu (Bupleuri Radix), Tian Hua Fen (Trichosanthis Radix), Dang Gui (Angelicae Sinensis Radix), Hong Hua (Carthami Flos), Gan Cao (Glycyrrhizae Radix Et Rhizoma), Chuan Shan Jia (Manis Squama), Da Huang (Rhei Radix Et Rhizoma), Tao Ren (Persicae Semen)	Grading of recommendation: B^*※*§^ Level of evidence: IIa [6]

Qi stagnation and blood stasis	Pathogenesis: Qi movement stagnation, static blood retention Principle: Activate blood and resolve stasis, move qi to relieve pain	Modified Ge Xia Zhu Yu Tang: Wu Ling Zhi (Faeces Trogopterpri), Dang Gui (Angelicae Sinensis Radix), Chuan Xiong (Chuanxiong Rhizoma), Tao Ren (Persicae Semen), Mu Dan Pi (Moutan Cortex), Chi Shao (Paeoniaeradix Rubra), Wu Yao (Linderae Radix), Yan Hu Suo (Corydalis Rhizoma), Gan Cao (Glycyrrhizae Radix Et Rhizoma), Xiang Fu (Cyperi Rhizoma), Hong Hua (Carthami Flos), Zhi Qiao (Aurantii Fructus) Modified Xue Fu Zhu Yu Tang: Tao Ren (Persicae Semen), Hong Hua (Carthami Flos), Dang Gui (Angelicae Sinensis Radix), Di Huang (Rehmanniae Radix), Niu Xi (Achyranthis Bidentatae Radix), Chuan Xiong (Chuanxiong Rhizoma), Jie Geng (Platycodonis Radix), Chi Shao (Paeoniaeradix Rubra), Zhi Qiao (Aurantii Fructus), Gan Cao (Glycyrrhizae Radix Et Rhizoma), Chai Hu (Bupleuri Radix)	Grading of recommendation: B^*※*§^ Level of evidence: IIa [7–10]

Combination of phlegm and blood stasis	Pathogenesis: Depressed gallbladder with harassing phlegm, static blood internal bind Principle: Regulate qi to resolve phlegm, activate blood to relieve pain	Modified Wen Dan Tang: Ban Xia (Pinelliae Rhizoma), Zhu Ru (Bambusae Caulis In Taenias), Zhi Shi (Aurantii Fructus Immaturus), Chen Pi (Citri Reticulatae Pericarpium Viride), Gan Cao (Glycyrrhizae Radix Et Rhizoma), Fu Ling (Poria) Modified Xue Fu Zhu Yu Tang: Tao Ren (Persicae Semen), Hong Hua (Carthami Flos), Dang Gui (Angelicae Sinensis Radix), Di Huang (Rehmanniae Radix), Niu Xi (Achyranthis Bidentatae Radix), Chuan Xiong (Chuanxiong Rhizoma), Jie Geng (Platycodonis Radix), Chi Shao (Paeoniaeradix Rubra), Zhi Qiao (Aurantii Fructus), Gan Cao (Glycyrrhizae Radix Et Rhizoma), Chai Hu (Bupleuri Radix) Modified Si Wu Tang plus Er Chen Tang: Dang Gui (Angelicae Sinensis Radix), Chi Shao (Paeoniaeradix Rubra), Chuan Xiong (Chuanxiong Rhizoma), Di Huang (Rehmanniae Radix), Chen Pi (Citri Reticulatae Pericarpium Viride), Fa Ban Xia (Pinelliae Rhizoma Praeparatum), Fu Ling (Poria), Gan Cao (Glycyrrhizae Radix Et Rhizoma), Hai Zao (Sargassum), Hong Hua (Carthami Flos), Xiang Fu (Cyperi Rhizoma), Mu Dan Pi (Moutan Cortex)	Grading of recommendation: B^*※*§^ Level of evidence: IIa [8–11]

Qi and blood deficiency	Pathogenesis: Dual deficiency of qi and blood, viscera and bowels with cachexia Principle: Tonify and harmonize the blood, resolve stasis to relieve pain	Modified Si Wu Tang: Shu Di Huang (Rehmanniae Radix Praeparata), Bai Shao (Paeoniae Radix Alba), Dang Gui (Angelicae Sinensis Radix), Chuan Xiong (Chuanxiong Rhizoma), Huang Qi (Astragali Radix), Bai Zhu (Atractylodis Macrocephalae Rhizoma)	Grading of recommendation: C^§^ Level of evidence: IV

**Table 6:** Herbal treatment on cancer constipation.

**Pattern**	**Pathogenesis and treatment principle**	**Recipe and ingredient herbs**	**Grading and level**
Qi and yin deficiency	Pathogenesis: Spleen-lung qi deficiency, fluid-humor deficiency Principle: Tonify the spleen and lung, enrich yin and increase humor	Modified Zeng Ye Tang: Xuan Shen (Scrophulariae Radix), Mai Dong (Ophiopogonis Radix), Di Huang (Rehmanniae Radix), Dang Gui (Angelicae Sinensis Radix), Yu Zhu (Polygonatiodoratirhizoma), Bei Sha Shen (Glehniae Radix), Huang Qi (Astragali Radix)	Grading of recommendation: B^*※*§^ Level of evidence: IIa [12–15]

Qi movement stagnation	Pathogenesis: Liver and spleen qi stagnation, bowel qi block Principle: Favor qi and remove food stagnation, direct qi downward to relax the bowels	Modified Liu Mo Tang: Bing Lang (Arecaesemen), Chen Xiang (Aquilariae Lignum Resinatum), Mu Xiang (Aucklandiae Radix), Wu Yao (Linderae Radix), Da Huang (Rhei Radix Et Rhizoma), Zhi Qiao (Aurantii Fructus)	Grading of recommendation: C^§^ Level of evidence: IV

Blood deficiency	Pathogenesis: Blood deficiency, intestines with cachexia Principle: Tonify blood and enrich yin, moisten dryness to relax the bowels	Modified Liu Mo Tang: Bing Lang (Arecaesemen), Chen Xiang (Aquilariae Lignum Resinatum), Mu Xiang (Aucklandiae Radix), Wu Yao (Linderae Radix), Da Huang (Rhei Radix Et Rhizoma), Zhi Qiao (Aurantii Fructus)	Grading of recommendation: C^§^ Level of evidence: IV

Yang deficiency	Pathogenesis: Yang deficiency and debilitation, yin and cold congealing and bind Principle: Tonify the kidney and warm yang, moisten the intestines to relax the bowels	Modified Ji Chuan Jian: Dang Gui (Angelicae Sinensis Radix), Niu Xi (Achyranthis Bidentatae Radix), Rou Cong Rong (Cistanches Herba), Ze Xie (Alismatis Rhizoma), Sheng Ma (Cimicifugae Rhizoma), Zhi Qiao (Aurantii Fructus)	Grading of recommendation: B^*※*§^ Level of evidence: IIa [16]

**Table 7:** Herbal treatment on cancer insomnia.

**Pattern**	**Pathogenesis and treatment principle**	**Recipe and ingredient herbs**	**Grading and level**
Liver-qi stagnation	Pathogenesis: Liver failing in free coursing, qi movement stagnation Principle: Soothe the liver and release the stagnation	Modified Chai Hu Shu Gan San: Chai Hu (Bupleuri Radix), Bai Shao (Paeoniae Radix Alba), Chen Pi (Citri Reticulatae Pericarpium Viride), Xiang Fu (Cyperi Rhizoma), Chuan Xiong (Chuanxiong Rhizoma), Zhi Qiao (Aurantii Fructus), Gan Cao (Glycyrrhizae Radix Et Rhizoma)	Grading of recommendation: C^§^ Level of evidence: IV

Liver depression and spleen deficiency	Pathogenesis: Liver depression and blood deficiency, spleen-stomach weakness Principle: Harmonize the liver and spleen, Soothe the liver and release the stagnation	Modified Xiao Yao San: Chai Hu (Bupleuri Radix), Dang Gui (Angelicae Sinensis Radix), Bai Shao (Paeoniae Radix Alba), Bo He (Menthae Haplocalycis Herba), Fu Ling (Poria), Sheng Jiang (Zingiberis Rhizoma Recens), Da Zao (Jujubae Fructus)	Grading of recommendation: C^§^ Level of evidence: IV

Qi and blood deficiency	Pathogenesis: Dual deficiency of qi and blood Principle: Tonify qi and replenish blood	Modified Ba Zhen Tang: Huang Qi (Astragali Radix), Fu Ling (Poria), Bai Zhu (Atractylodis Macrocephalae Rhizoma), Dang Gui (Angelicae Sinensis Radix), Chuan Xiong (Chuanxiong Rhizoma), Shu Di Huang (Rehmanniae Radix Praeparata), Dang Shen (Codonopsis Radix), Yu Jin (Curcumae Radix), Mu Xiang (Aucklandiae Radix), Suan Zao Ren (Ziziphispinosaesemen), Yuan Zhi (Polygalae Radix), Zhi Gan Cao (Glycyrrhizae Radix Et Rhizoma Praeparata Cum Melle)	Grading of recommendation: B^*※*§^ Level of evidence: IIa [17, 18]

Qi and yin deficiency	Pathogenesis: Dryness damaging the lung and stomach, fluid-humor depletion Principle: Tonify the lung and stomach, engender fluid and moisten dryness	Modified Si Jun Zi Tang plus Sha Can Mai Dong Tang: Huang Qi (Astragali Radix), Bai Zhu (Atractylodis Macrocephalae Rhizoma), Bei Sha Shen (Glehniae Radix), Tian Dong (Asparagi Radix), Mai Dong (Ophiopogonis Radix), Gua Lou Pi (Trichosanthis Pericarpium), Shi Shang Bai (Selaginella Doederleinii Hieron.), Shi Jian Chuan (Salvia Chinensia Benth.), Bai Hua She She Cao (Spreading Hedyotis Herb), Chan Pi (Bufo Melanostictus Schneider), Xia Ku Cao (Prunellae Spica), Mu Li (Ostreae Concha)	Grading of recommendation: B^*※*§^ Level of evidence: IIa [19]

Heart and spleen deficiency	Pathogenesis: Heart blood deficiency, spleen qi deficiency Principle: Tonify qi and fortify the spleen, nourish the heart to tranquilize	Modified Ren Shen Gui Pi Tang: Ren Shen (Ginseng Radix Et Rhizoma), Bai Zhu (Atractylodis Macrocephalae Rhizoma), Huang Qi (Astragali Radix), Dang Gui (Angelicae Sinensis Radix), Yuan Zhi (Polygalae Radix), Suan Zao Ren (Ziziphispinosaesemen), Fu Shen (Tuckahoe With Pine), Mu Xiang (Aucklandiae Radix), Long Yan Rou (Longan Arillus), Sheng Jiang (Zingiberis Rhizoma Recens), Da Zao (Jujubae Fructus), Gan Cao (Glycyrrhizae Radix Et Rhizoma)	Grading of recommendation: C^§^ Level of evidence: IV

**Table 8:** Acupuncture on cancer pain.

**Pattern**	**Acupuncture point**	**Grading and level**
Main acupoints for all cancer pain patterns: He Gu (LI4), Tai Chong (LR3), A Shi points		Grading of recommendation: B^*※*§^ Level of evidence: IIa [20–26]

Qi movement stagnation	Nei Guan (PC6), Gong Sun (SP4), Qi Men (LR14), Zhong Wan (RN12), Zhi Gou (SJ6)	Grading of recommendation: C^§^ Level of evidence: IV

Phlegm-dampness congealing	Zu San Li (ST36), Feng Long (ST40), Yin Ling Quan (SP9)	Grading of recommendation: C^§^ Level of evidence: IV

Static blood obstructing	Xue Hai (SP10), Ge Shu (BL17), Dan Zhong (RN17), San Yin Jiao (SP6), Nei Guan (PC6)	Grading of recommendation: C^§^ Level of evidence: IV

Qi stagnation and blood stasis	Nei Guan (PC6), Xue Hai (SP10), Ge Shu (BL17), Liang Qiu (ST34)	Grading of recommendation: C^§^ Level of evidence: IV

Combination of phlegm and blood stasis	Feng Long (ST40), Xue Hai (SP10), Zu San Li (ST36), San Yin Jiao (SP6), Nei Guan (PC6)	Grading of recommendation: C^§^ Level of evidence: IV

Qi and blood deficiency	Qi Hai (RN6), Zhong Wan (RN12), Zu San Li (ST36), Xue Hai (SP10), Pi Shu (BL20), Fei Shu (BL13), San Yin Jiao (SP6)	Grading of recommendation: C^§^ Level of evidence: IV

**Table 9:** Acupuncture on cancer constipation.

**Pattern**	**Acupuncture point**	**Grading and level**
Main acupoints for all cancer constipation patterns: Tian Shu (ST25), Zu San Li (ST36), Shang Ju Xu (ST37), Da Chang Shu (BL25), Zhi Gou (SJ6)		Grading of recommendation: B^*※*§^ Level of evidence: IIa [12] Grading of recommendation: C^§^ Level of evidence: IV

Qi and yin deficiency	Tai Xi (KI3), Yin Ling Quan (SP9), San Yin Jiao (SP6), Xue Hai (SP10), Tong Li (HT5), Pi Shu (BL20), Shen Shu (BL23)	Grading of recommendation: C^§^ Level of evidence: IV

Qi movement stagnation	Zhong Wan (RN12), Xing Jian (LR2)	Grading of recommendation: C^§^ Level of evidence: IV

Yang deficiency	Shen Que (RN8), Qi Hai (RN6)	Grading of recommendation: C^§^ Level of evidence: IV

**Table 10:** Acupuncture on cancer insomnia.

**Pattern**	**Acupuncture point**	**Grading and level**
Main acupoints for all cancer insomnia patterns: Shen Men (HT7), San Yin Jiao (SP6), Bai Hui (DU20)		Grading of recommendation: B^*※*§^ Level of evidence: IIa [27, 28]

Heart and spleen deficiency	Xin Shu (BL15), Jue Yin Shu (BL14), Pi Shu (BL20)	Grading of recommendation: C^§^ Level of evidence: IV

## Data Availability

Details of data mining, selection, extraction, and assessment carried out to support the findings of this study are available from the corresponding author upon request

## References

[1] The Hong Kong Cancer Registry (HKCaR) Overview of Hong Kong Cancer Statistics of 2015. http://www3.ha.org.hk/cancereg/pdf/overview/Summary%20of%20CanStat%202015.pdf/.2.

[2] Sham M. K. M. (2003). Pain Relief and Palliative Care in Hong Kong. *Journal of Pain and Palliative Care Pharmacotherapy*.

[3] Laugsand E. A., Kaasa S., De Conno F., Hanks G., Klepstad P. (2009). Intensity and treatment of symptoms in 3,030 palliative care patients: A cross-sectional survey of the EAPC Research Network. *Journal of Opioid Management*.

[4] Tsai J., Wu C., Chiu T., Hu W., Chen C. (2006). Symptom patterns of advanced cancer patients in a palliative care unit. *Palliative Medicine*.

[5] The Hospital Authority https://www.ha.org.hk/chinesemedicine/resources/ICWM.pdf/.

[6] Li H.-D., Ge X., Deng Q., Huang M., Xie J.-X. (2010). Tao Hong Si Wu Tang and MVP Chemotherapy in Treatment of Haemostasis Inter-junction Esophageal Cancer. *Medical Recapitulate*.

[7] Wang M., Chen Q. (2013). Gexia Zhuyu Decoction Combined with Shixiaosan Combined Three Ladder Analgesic Treatment of Qi Stagnation and Blood Stasis Type Tumors in Advanced Cancer Patients Randomized Parallel Controlled Study. *Journal of Practical Traditional Chinese Internal Medicine*.

[8] Chen H., He S., Liu S. (2014). Clinical Observation of 60 Cases of Xue Fu Zhu Yu Decoction Combined with Morphine Sulfate Sustained-release Tablets in Treatment of Advanced Cancer Pain. *Guiding Journal of Traditional Chinese Medicine and Pharmacy*.

[9] Fan D., Luo Y. (2014). Xuefu Zhuyu Decoction Combined with Fortanodyn Treating Blood Stasis Type of Cancer Pain. *Journal of Practical Traditional Chinese Internal Medicine*.

[10] Qiao X., Zhao J. (2012). 60 Cases of Xue Fu Zhu Yu Decoction in Treatment of Cancer Pain Patients with Blood Stasis Stagnation Syndrome. *Guangming Journal of Chinese Medicine*.

[11] Zhang L., Ma J., Sun W., Zhang H. (2009). Clinical Observation of Wendan Decoction in Collaborative Treatment of Advanced Cancer Pain with Phlegm-dampness Syndrome. *Xinjiang Journal of Traditional*.

[12] Chen C.-M., Lin L.-Z., Zhang E.-X. (2014). Standardized treatment of chinese medicine decoction for cancer pain patients with opioid-induced constipation: A multi-center prospective randomized controlled study. *Chinese Journal of Integrative Medicine*.

[13] Li J.-Y., Fang Z.-X., Song L., Lv Y., Chen M.-X. (2013). Clinical curative effects of Extra-large Chengqi Decoction in treating bowel dryness and fluid deficiency type constipation caused by opium analgesics. *Journal of Traditional Chinese Medicine University of Hunan*.

[14] Li K. (2013). Clinical Observation of Qi Shu Zeng Ye Decoction in Treatment of Constipation Induced by Opioid. *Jilin Journal of Traditional Chinese Medicine*.

[15] Zhenxiong H., Jianxiao J. (2007). Clinical Observation of Yi Qi Zeng Ye Decoction in Treatment of Advanced Cancer Constipation. *Journal of Practical Traditional Chinese Medicine*.

[16] Gao H., Yin D., Zhang N. (2007). Clinical Observation of Modified Ji Chuan Jian in Treatment of Cancer Constipation Patients. *Liaoning Journal of Traditional Chinese Medicine*.

[17] Jianhong H., Jun C., Yurong X. (2013). Bazhen Decoction in Treatment of Postoperative Breast Cancer Patients after Chemotherapy Curative Effect of Insomnia. *Guangming Journal of Chinese Medicine*.

[18] Pan G., Wang S., Yan W., Yu L., Li Y. (2014). Effect of the TCM Syndrome Differentiation and Treatment on Progression-free Survival Time and Quality of Life of Postoperative Colorectal Cancer Patients. *Henan Traditional Chinese Medicine*.

[19] Hu H.-J., Liu L.-S., Shen L.-P. (2014). Clinical Research on Therapeutic Effect of Strengthening Body Resistance TCM Consolidated Therapy for Advanced Non-Small-Cell Lung Cancer. *Journal of Chengdu University of Traditional Chinese Medicine*.

[20] Paley C. A., Johnson M. I., Tashani O. A., Bagnall A.-M. (2011). Acupuncture for cancer pain in adults. *Cochrane Database of Systematic Reviews*.

[21] Choi T.-Y., Lee M. S., Kim T.-H., Zaslawski C., Ernst E. (2012). Acupuncture for the treatment of cancer pain: a systematic review of randomised clinical trials. *Supportive Care in Cancer*.

[22] Peng H., Peng H.-D., Xu L., Lao L. X. (2010). Efficacy of acupuncture in treatment of cancer pain: a systematic review. *Journal of Chinese Integrative Medicine/Zhong Xi Yi Jie He Xue Bao*.

[23] Duan K.-N., Ma W.-H., Meng M.-B. (2009). Traditional Medicine plus 3-step Analgesic Ladder for Cancer Pain:A Meta-analysis of Randomized Controlled Trial. *West China Medical Journal*.

[24] Tan G. S., Lin Z. T., Wang Q. (2012). Clinical observation on analgesia effect in cancer pain treated with acupuncture and Western medicine. *World Journal of Integrated Traditional and Western Medicine*.

[25] Kang F.-H. (2013). Clinical observation of acupuncture combined with xaoliu zhitong decoction in the treatment of intermediate and advanced stage cancerous pain. *Journal of Hunan University of Chinese Medicine*.

[26] Feng J., Kong D. (2011). The Comparative Efficacy of the Integrated Traditional Chinese Medicine and Western Medicine Treat Cancer pain in end Stage. *Liaoning Journal of Traditional Chinese Medicine*.

[27] Feng Y., Wang X.-Y., Li S.-D. (2011). Clinical research of acupuncture on malignant tumor patients for improving depression and sleep quality. *Journal of Traditional Chinese Medicine*.

[28] Shi M., Han K. E., Xia Y. (2013). Study about Soothing Liver and Reinforcing Essence of Kidney by Acupuncture in Adjusting Depression of Gynecological Malignancies after Surgery and Chemotherapy. *Journal of Liaoning University of Traditional Chinese Medicine*.

[29] Shi N., Zhong L. L. D., Han X. (2015). Enhanced Evidence-Based Chinese Medicine Clinical Practice Guidelines in Hong Kong: A Study Protocol for Three Common Diseases. *Evidence-Based Complementary and Alternative Medicine*.

[30] Zheng G., Jiang M., He X. (2011). Discrete dervative: adata slicing algorithm for exploration of sharing biological networks between rheumatoid arthritis and coronary heart disease. *BioData Mining*.

[31] Liu J. P. (2007). The composition of evidence body of traditional medicine and recommendations for its evidence grading. *Zhongguo Zhong Xi Yi Jie He Za Zhi*.

[32] Burton A. W., Chai T., Smith L. S. (2014). Cancer pain assessment. *Current Opinion in Supportive and Palliative Care*.

[33] Goudas L. C., Bloch R., Gialeli-Goudas M., Lau J., Carr D. B. (2005). The epidemiology of cancer pain. *Cancer Investigation*.

[34] Deandrea S., Corli O., Consonni D., Villani W., Greco M. T., Apolone G. (2014). Prevalence of breakthrough cancer pain: A systematic review and a pooled analysis of published literature. *Journal of Pain and Symptom Management*.

[35] Portenoy R. K. (2011). Treatment of cancer pain. *The Lancet*.

[36] Bao Y., Kong X., Yang L. (2014). Complementary and Alternative Medicine for Cancer Pain: An Overview of Systematic Reviews. *Evidence-Based Complementary and Alternative Medicine*.

[37] Wang J. Y., Xu L., Zhang R. X., Lao L. (2011). Traditional Chinese medicine for cancer pain. *Zhong Xi Yi Jie He Xue Bao*.

[38] Xiangyong Y., Zhongsheng Y., Wenchao L. (2016). External application of traditional Chinese medicine in the treatment of bone cancer pain: a meta-analysis. *Supportive Care in Cancer*.

[39] Vinjamury S. P., Li J. T., Hsiao E. (2013). Effects of acupuncture for cancer pain and quality of life - a case series. *Chinese Medicine*.

[40] Zhu S.-J., Jia L.-Q., Li P.-W. (2011). Clinical evaluation of the efficacy of external therapies of traditional Chinese medicine in treatment of cancer pain. *Journal of Chinese Integrative Medicine*.

[41] Xu L., Li X. L., Ge A., Yu S., Li J., Mansky P. J. (2007). Chinese herbal medicine for cancer pain. *Integrative Cancer Therapies*.

[42] Cai P., Li L., Hong H. (2018). A Chinese medicine warm compress (Wen Jing Zhi Tong Fang), combined with WHO 3-step analgesic ladder treatment for cancer pain relief. *Medicine*.

[43] Potter J., Hami F., Bryan T., Quigley C. (2003). Symptoms in 400 patients referred to palliative care services: Prevalence and patterns. *Palliative Medicine*.

[44] Curtis E. B., Krech R., Walsh T. D. (1991). Common symptoms in patients with advanced cancer. *J Palliat Care*.

[45] Fallon M. T. (1999). Constipation in cancer patients: Prevalence, pathogenesis, and cost- related issues. *European Journal of Pain*.

[46] Rao S. S., Rattanakovit K., Patcharatrakul T. (2016). Diagnosis and management of chronic constipation in adults. *Nature Reviews Gastroenterology & Hepatology*.

[47] Larkin P., Sykes N., Centeno C. (2008). The management of constipation in palliative care: clinical practice recommendations. *Palliative Medicine*.

[48] Cheng C. W., Kwok A. O. L., Bian Z. X., Tse D. M. W. (2013). A cross-sectional study of constipation and laxative use in advanced cancer patients: Insights for revision of current practice. *Supportive Care in Cancer*.

[49] Cheng C.-W., Kwok A. O. L., Bian Z.-X., Tse D. M. W. (2012). The Quintessence of Traditional Chinese Medicine: Syndrome and Its Distribution among Advanced Cancer Patients with Constipation. *Evidence-Based Complementary and Alternative Medicine*.

[50] Smith M. E., Bauer-Wu S. (2012). Traditional Chinese Medicine for Cancer-Related Symptoms. *Seminars in Oncology Nursing*.

[51] Chung V. C., Wu X., Lu P. (2016). Chinese Herbal Medicine for Symptom Management in Cancer Palliative Care: Systematic Review And Meta-analysis. *Medicine (Baltimore)*.

[52] Lu W., Rosenthal D. S. (2013). Acupuncture for cancer pain and related symptoms.. *Current Pain and Headache Reports*.

[53] Zhao W. H., Su Z. X., Cao X. M. (2006). The effect of Qing-Shu Particles in the Treatment on Constipation resulted from Chemotherapy to the Lymphoma Patients. *Modern Oncology*.

[54] Lau C. H., Wu X., Chung V. C. (2016). Acupuncture and Related Therapies for Symptom Management in Palliative Cancer Care: Systematic Review and Meta-Analysis. *Medicine (Baltimore)*.

[55] Yu C. T., Ko N. Y. (2010). Evidence-based nursing care for cancer patients with opioid-induced constipation. *Hu Li Za Zhi*.

[56] Berger A. M., Farr L. A., Kuhn B. R., Fischer P., Agrawal S. (2007). Values of Sleep/Wake, Activity/Rest, Circadian Rhythms, and Fatigue Prior to Adjuvant Breast Cancer Chemotherapy. *Journal of Pain and Symptom Management*.

[57] Palesh O. G., Roscoe J. A., Mustian K. M. (2010). Prevalence, demographics, and psychological associations of sleep disruption in patients with cancer: University of Rochester cancer center-community clinical oncology program. *Journal of Clinical Oncology*.

[58] Savard J., Ivers H., Villa J., Caplette-Gingras A., Morin C. M. (2011). Natural course of insomnia comorbid with cancer: an 18-month longitudinal study. *Journal of Clinical Oncology*.

[59] Savard J., Simard S., Blanchet J., Ivers H., Morin C. M. (2001). Prevalence, clinical characteristics, and risk factors for insomnia in the context of breast cancer. *SLEEP*.

[60] Berger A. M., Grem J. L., Visovsky C., Marunda H. A., Yurkovich J. M. (2010). Fatigue and Other Variables During Adjuvant Chemotherapy for Colon and Rectal Cancer. * Oncology Nursing Forum*.

[61] Davidson J. R., MacLean A. W., Brundage M. D., Schulze K. (2002). Sleep disturbance in cancer patients. *Social Science & Medicine*.

[62] Sela R. A., Watanabe S., Nekolaichuk C. L. (2005). Sleep disturbances in palliative cancer patients attending a pain and symptom control clinic.. *Palliative & supportive care.*.

[63] Mercadante S., Girelli D., Casuccio A. (2004). Sleep disorders in advanced cancer patients: Prevalence and factors associated. *Supportive Care in Cancer*.

[64] Koopman C., Nouriani B., Erickson V. (2002). Sleep disturbances in women with metastatic breast cancer. *The Breast Journal*.

[65] Le Guen Y., Gagnadoux F., Hureaux J. (2007). Sleep disturbances and impaired daytime functioning in outpatients with newly diagnosed lung cancer. *Lung Cancer*.

[66] Romito F., Cormio C., De Padova S. (2014). Patients attitudes towards sleep disturbances during chemotherapy. *European Journal of Cancer Care*.

[67] Savard J., Morin C. M. (2001). Insomnia in the context of cancer: A review of a neglected problem. *Journal of Clinical Oncology*.

[68] Kim W., Lee W.-B., Lee J.-W. (2015). Traditional herbal medicine as adjunctive therapy for breast cancer: a systematic review. *Complementary Therapies in Medicine*.

[69] Haddad N. E., Palesh O. (2014). Acupuncture in the treatment of cancer-related psychological symptoms. *Integrative Cancer Therapies*.

[70] Tao W., Luo X., Cui B. (2015). Practice of traditional Chinese medicine for psycho-behavioral intervention improves quality of life in cancer patients: a systematic review and meta-analysis. *Oncotarget *.

[71] Carmady B., Smith C. A. (2011). Use of Chinese medicine by cancer patients: a review of surveys. *Chinese Medicine*.

[72] NIH Consensus Conference (1998). Acupuncture. *The Journal of the American Medical Association*.

[73] China Academy of Chinese Medical Sciences (2011). *Evidence-based Guidelines of Clinical Practice in Chinese Medicine Internal Medicine*.

